# Association Between Obesity and Falls Among Korean Adults: A Population-Based Cross-Sectional Study

**DOI:** 10.1097/MD.0000000000003130

**Published:** 2016-03-25

**Authors:** So Young Kim, Min-Su Kim, Songyong Sim, Bumjung Park, Hyo Geun Choi

**Affiliations:** From the Department of Otorhinolaryngology—Head & Neck Surgery and Cancer Research Institute (SYK, M-SK), Seoul National University College of Medicine, Seoul, Korea; Department of Otorhinolaryngology—Head and Neck Surgery (SYK), CHA Bundang Medical Center, CHA University; Department of Otorhinolaryngology-Head and Neck Surgery (M-SK), Korea University Ansan Hospital, Korea University; Department of Statistics (SS), Hallym University, Chuncheon, Korea; and Department of Otorhinolaryngology—Head & Neck Surgery (BP, HGC), Hallym University Sacred Heart Hospital, Anyang, Korea.

## Abstract

The objective of this study was to evaluate the association between falls and obesity using Asian body mass index (BMI) classifications. Using the data from the Korean community health survey in 2011, a total of 229,226 participants ranging from 19 to 106 years old were included in this study. The BMI groups were classified as underweight (<18.5), healthy (18.5 ≤ BMI < 23), overweight (23 ≤ BMI <25), and obese (≥25) using Asian BMI classifications. The associations between BMI groups and falls (≥1 time or ≥2 times per year) were analyzed using multiple logistic regression analyses with complex sampling. A subgroup analysis was conducted according to age (19–40, 41–60, and ≥61 years) and the location of the fall (indoor and outdoor). Physical activity, household income, education level, alcohol consumption, smoking, stress level, and medical comorbidities were adjusted as confounders. In total, 16.8% and 6.1% of the participants experienced falls ≥1 time and ≥2 times per year, respectively. Compared to the healthy weight group, the other BMI groups showed a significant U-shaped relationship with falls ≥1 time (AOR underweight = 1.12, 95% CI [confidence interval] = 1.05–1.19; AOR obese = 1.06, 95% CI = 1.02–1.10, *P* < 0.001) and ≥2 times (AOR underweight = 1.14, 95% CI = 1.04–1.26; AOR obese = 1.04, 95% CI = 0.99–1.10, *P* < 0.001). Obese status was significantly associated with falls (≥1 fall per year) in all age groups, whereas being underweight was significantly associated with falls in the 19 to 40 year age group only. In conclusion, both underweight and obese statuses were significantly associated with falls in this adult Korean population. However, the relationship between BMI group and falls varied according to age and the location of the falls.

## INTRODUCTION

Falls are common accidental injuries, accounting for approximately 80% of disabilities from unintentional injuries, with the exception of traffic accidents.^1^ This high incidence of falls leads to enormous medical and economic costs.^2^ In particular, subjects who are susceptible to fall-related injuries, such as the elderly, can sustain serious injuries from falls. Moreover, the recent improvement of medical care has increased in this elderly population. Therefore, many studies have investigated related factors and preventative methods for falls, focusing primarily on the elderly.^3–5^ However, there has been a paucity of concern regarding falls in young adults, which may also cause a severe enough loss of physical and social abilities to interfere with daily functioning.

Numerous factors are associated with falls. These factors can be categorized as intrinsic and extrinsic factors.^6^ Extrinsic factors refer to environmental factors such as medication effects or home hazards, whereas intrinsic factors reflect the functional and health status of the subjects including age, balance function, muscle weakening, cognitive abilities, orientation, visual impairment, fear of falls, and obesity.^7^ Sedentary behavior and sedentary occupation are risk factors for obesity and metabolic syndrome, and inadequate physical training can lead to falls.^8,9^ Among these various factors, obesity has received attention because of its increasing prevalence and adverse metabolic and other health-related outcomes. Previous studies have reported a positive correlation between falls and indicators of obesity, primarily body mass index (BMI) or visceral fat.^10–13^ However, there may be ethnic variations affecting the relationship between the proportion of body fat and BMI; compared to Caucasians, Asians showed increased body fat content at identical BMIs.^14,15^ Therefore, the BMI classification of Asians is different from the World Health Organization (WHO) BMI classifications: underweight, <18.5 kg/m^2^; healthy, 18.5 ≤ BMI < 23 kg/m^2^; overweight, 23 ≤ BMI < 25 kg/m^2^; and obese, ≥25 kg/m^2^.^16^ However, no studies have investigated the association between obesity and falls in Asians using Asian BMI classifications.

The majority of previous studies have focused on the relationship between obese status and falls; ^10,11,13^ however, the underweight condition is also associated with disproportional body composition, diminished mobility, and reduced stability, all of which may have detrimental effects on falls.^17^ Numerous studies have investigated the relationship between falls and obese groups, whereas few studies have been conducted in underweight groups. Therefore, the present study was performed to analyze the association of falls with a wide range of BMI groups, from underweight through obese, based on the Asian BMI classifications. Furthermore, differences by age and the location of the falls were analyzed. To our knowledge, this is the first study on the association between falls and a wide range of BMI groups in a large representative population.

## METHODS

### Study Population and Data Collection

This study was approved by the Institutional Review Board of the Korea Centers for Disease Control and Prevention (IRB no. 2011–05CON-04-C). Written informed consents were obtained from all of the participants prior to the survey.

This study was a cross-sectional study that used data from the Korean Community Health Survey (KCHS). Data from the KCHS that was conducted in 2011 were analyzed. The data were collected by the Centers for Disease Control and Prevention of Korea. The survey gathered information through face-to-face, paper-assisted personal interviews that were conducted by trained interviewers. The sample size for the KCHS was 900 subjects in each of the 253 community units, which included 16 metropolitan cities and provinces. The KCHS uses a 2-stage sampling process. In the first stage, a sample area is selected (tong/ban/ri) as the primary sample unit; this area is selected according to the number of households in the area using a probability proportional to the sample area. In the second stage, a household directory is created by identifying the number of households in the selected sample tong/ban/ri. Sample households are then selected using systematic sampling methods. This process is used to ensure that the sample units are representative of the entire population.^18^ For the sample to be statistically representative of the population, the survey is weighted by statisticians based on the data collected.^19^

**FIGURE 1:**
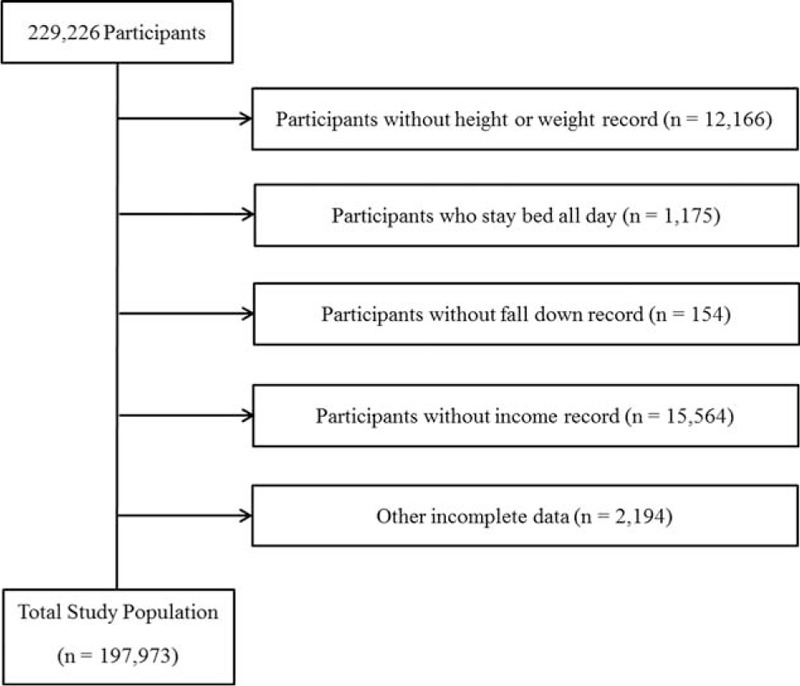
A schematic of participant selection in the present study. Individuals with incomplete survey data were excluded from the study; complete data were available and analyzed for 197,973 of the 229,226 total participants.

Of a total of 229,226 participants ranging from 19 to 106 years old, we excluded the following participants from this study: participants who did not fill out weight or height information (12,166 participants); participants who stayed in bed all day (1175 participants); participants who lacked slip or fall records (154 participants); participants without income records (15,564 participants); and participants who had incomplete data regarding history of exercise, education level, smoking, alcohol consumption, sleep hours, stress levels, diabetes, cerebral stroke, angina or myocardial infarction, or arthritis (2194 participants). After excluding these participants, a total of 197,973 participants were included in this study (Figure 1).

## SURVEY

Some variables were treated as the same way of our previous studies.^20–23^ To measure physical activity, the participants were asked about the number of days they engaged in vigorous exercise with considerable shortness of breath for >10 minutes in the last week and the number of days they engaged in moderate exercise with slight shortness of breath for >10 minutes in the last week.^21^ Monthly income was separated into lowest, low-middle, upper-middle, and highest quartiles,^20–23^ based on methods recommended by the Organization for Economic Cooperation and Development ^24^ (dividing household income by the square root of the number of household members). To explore the influence of the education level, the “low” education group was defined as uneducated participants and those who had graduated only from elementary or middle schools, whereas high school comprised the “middle” group, and junior college graduates, graduate school, and college graduates were assigned to the “high” group.^21^ Alcohol consumption was divided into 4 groups: none, ≤1 time a month, 2 to 4 times a month, and ≥2 times a week.^20,21^ Smoking status was classified as nonsmoker, past smoker, and current smoker.^21^ Past smokers who had quit smoking <1 year previously were considered as the current smoker group.^21^ The participants were asked whether they usually feel stressed, and stress levels were grouped into 4 categories from no stress to severe stress.^21^ Amount of sleep was divided into the following categories: ≤5 hours per day, 6 hours per day, 7 hours per day, 8 hours per day, and ≥9 hours per day.^21,23^ The participants who slept <3 hours or >12 hours were excluded from this study.^21,23^

The participants were asked about their history of other comorbidities, such as diabetes, stroke, angina or myocardial infarction, and arthritis. Those who reported a history of any of these diseases as diagnosed by a medical doctor were recorded as positive.

Participants <110 cm or 30 kg were excluded in this study. Based on the international classification of adult underweight, overweight, and obese BMI values according to WHO,^25^ participants were classified into 4 BMI groups: underweight, <18.5; healthy, 18.5 ≤ BMI < 25; overweight, 25 ≤ BMI <30; and obese, ≥30. However, based on this BMI classification, only 2.1% of our study population was classified as obese. Moreover, because of the different proportions of adipose tissue between Caucasians and Asians, previous studies have recommended different reference values for BMI classifications in Asians: underweight, <18.5; healthy, 18.5 ≤ BMI < 23; overweight, 23 ≤ BMI < 25; and obese, ≥25.^16^ Therefore, we classified the BMI groups according to these Asian reference values.

The participants were asked “how many times did you slip or fall in the past year?” The participants who had a history of slips or falls ≥1 time per year were recorded as positive.^21^ Because we believed that anyone could slip or fall once just by chance regardless of a physical condition, a secondary analysis was conducted using the history of slip or falls ≥2 times per year.^21^ For the subgroup analysis according to their slip or fall site, the fall sites were divided into indoors (washroom, bedroom, living room, kitchen, floor, or other inside) or outdoors (farming or fishing area, transport area, exercise place, business district, or other outer area).^21^ In this subgroup analysis, the participants who fell indoors or outdoors ≥1 time per year were recorded as positive.^21^

### Statistical Analysis

Differences in mean age, days of vigorous exercise, and days of moderate exercise between normal participants (control) and fall participants were compared using a linear regression analysis with complex sampling. The rate differences of sex, diabetes, stroke, angina or myocardial infarction, arthritis, asthma history, income level, education level, smoking, alcohol consumption, sleep hours, stress level, and BMI were compared using the chi-square test with the Rao-Scott correction.

The associations between slips and falls (yes or no) and obesity groups were analyzed using the following methods: a simple logistic regression analysis with complex sampling (unadjusted); a multiple logistic regression analysis with complex sampling adjusted for age and sex (model 1); and a multiple logistic regression analysis with complex sampling adjusted for age, sex, days of vigorous exercise, days of moderate exercise, income level, education level, smoking, alcohol consumption, sleep hours, stress level, diabetes, cerebral stroke, angina or myocardial infarction, and arthritis history (model 2).

For the subgroup analysis according to age, age was divided into 3 groups: 19 to 40 years (n = 62,396); 41 to 60 years (n = 79,656); and 61+ years (n = 55,921). A subgroup analysis of obesity for falls (≥1 time or ≥2 times per year) was performed using a multiple logistic regression analysis with complex sampling (model 2).

To analyze the participants according their fall sites, a subgroup analysis of obesity for falls (indoor or outdoor) using a multiple logistic regression analysis with complex sampling was performed (model 2).

Two-tailed analyses were conducted, and *P* < 0.05 was considered to indicate significance. The adjusted odds ratios (AOR) and 95% confidence interval (CI) for falls were calculated. All of the results are presented as weighted values. The results were analyzed statistically using SPSS version 21.0 (IBM, Armonk, NY). The data were analyzed on January 5, 2016.

## RESULTS

In total, 16.8% (33,236/164,737) of the participants experienced falls ≥1 time per year, and 6.1% (11,989/185,984) of the participants experienced falls ≥2 times per year (Table 1). Specifically, there were 21,247 (10.7%), 7287 (3.7%), 2730 (1.4%), 523 (0.3%), and 1458 (0.7%) participants who experienced falls 1, 2, 3, 4, and ≥5 times per year, respectively. The average BMI was 23.0 ± 3.0, and there were 92,081 (46.5%), 11,017 (5.6%), 48,047 (24.3%), and 46,828 (23.7%) participants who were classified as healthy, underweight, overweight, and obese, respectively, based on BMI.

**TABLE 1:**
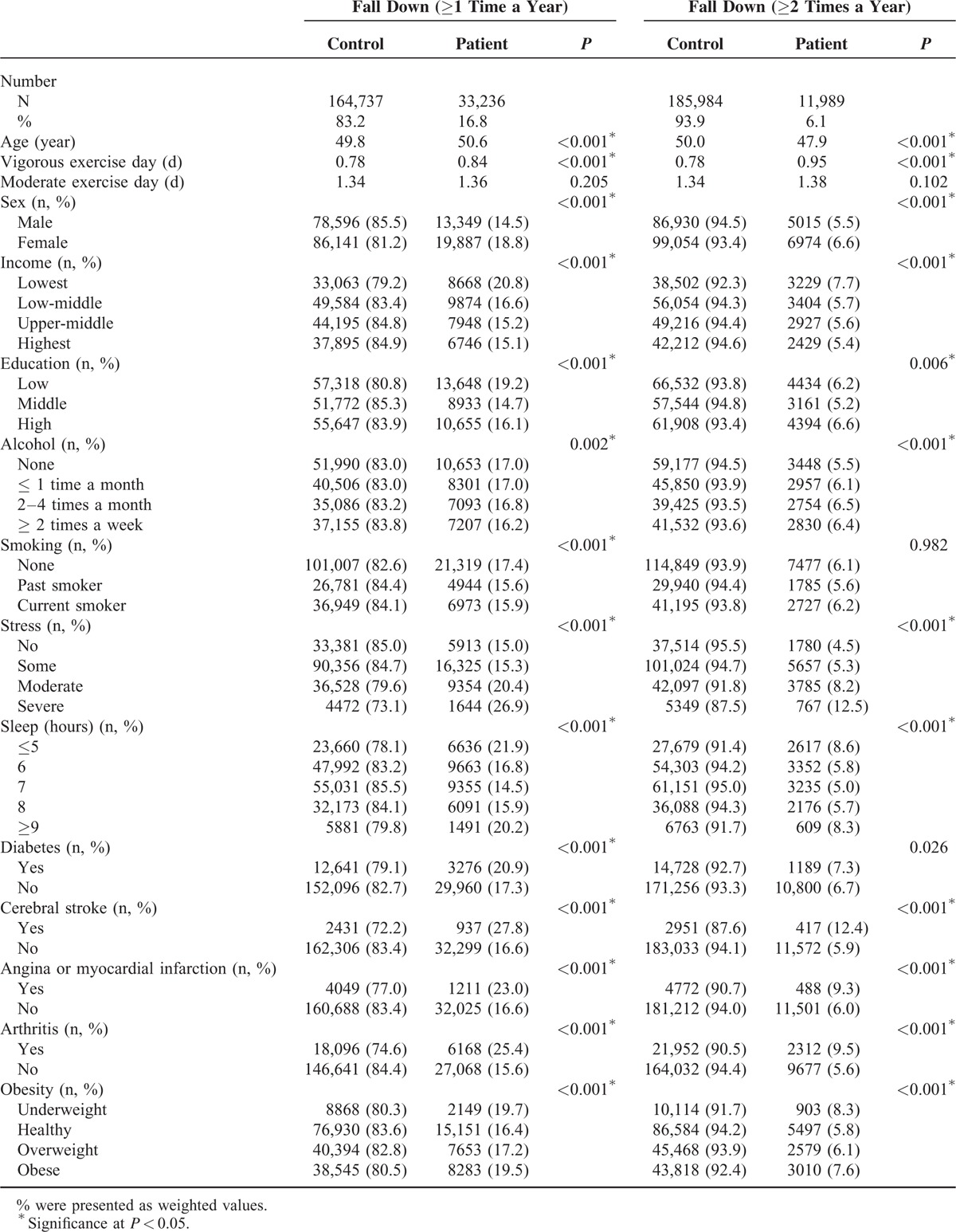
General Characteristics of Participants

The proportions of participants in the BMI groups were different between the fall and nonfall groups (*P* < 0.001; Table 1). In addition, most of the collected variables were significantly different according to the experience of the falls. Old age, many days of vigorous exercise, female sex, low income, low education level, heavy alcohol, high stress level, inadequate sleep duration, and medical comorbidities including diabetes, stroke, angina or myocardial infarction, and arthritis showed significant differences in terms of falls (each *P* < 0.05). Thus, all of these variables were considered as covariates in the subsequent logistic regression analyses.

Compared to the healthy weight groups, the underweight and obese groups showed a significant U-shaped relationship with falls (Table 2): placement in the underweight and obese groups was positively correlated with number of falls in the unadjusted, model 1, and model 2 analyses. The AOR for falls ≥1 time per year was 1.12 for the underweight group (95% CI = 1.05–1.19) and 1.06 for the obese group (95% CI = 1.02–1.10, *P* < 0.001) (model 2). This U-shaped relationship between BMI groups and falls was maintained for individuals who fell ≥2 times per year. Both the underweight and obese groups showed significant associations with falls ≥2 times per year (AOR underweight = 1.14, 95% CI = 1.04–1.26; AOR obese = 1.04, 95% CI = 0.99–1.10, *P* < 0.001). However, there was no significant correlation between placement in the overweight group and either ≥1 or ≥2 falls per year.

**TABLE 2:**
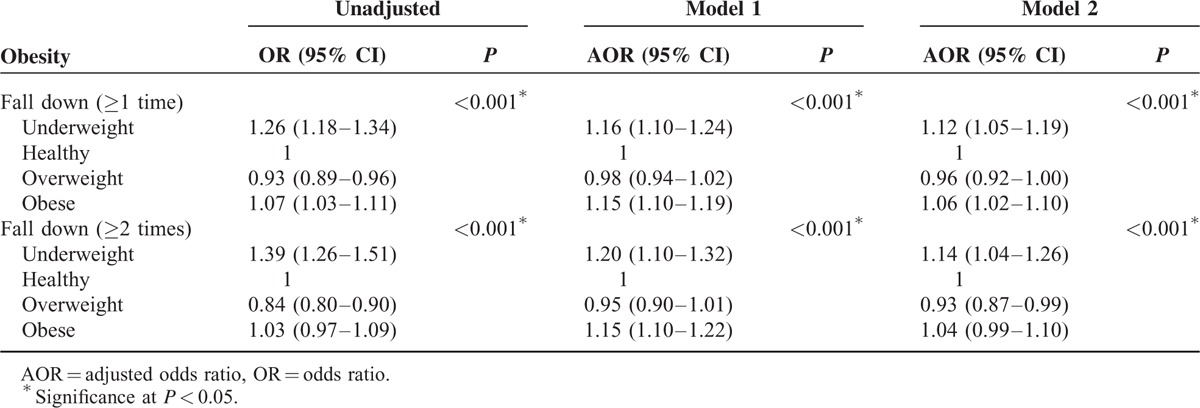
Odds Ratios of Obesity for Fall Down (≥1 Time or ≥2 Times a Year) Using Simple and Multiple Logistic Regression Analyses With Complex Sampling

In the subgroup analysis according to age, the obese group showed significant correlations with falls (≥1 and ≥2 falls per year) for all age groups, except the 41- to 60-year age group (≥2 falls per year) (Table 3). Underweight status was significantly associated with ≥1 fall per year in only the 19- to 40-year age group (AOR = 1.09, 95% CI = 1.00–1.18, *P* < 0.001).

**TABLE 3:**
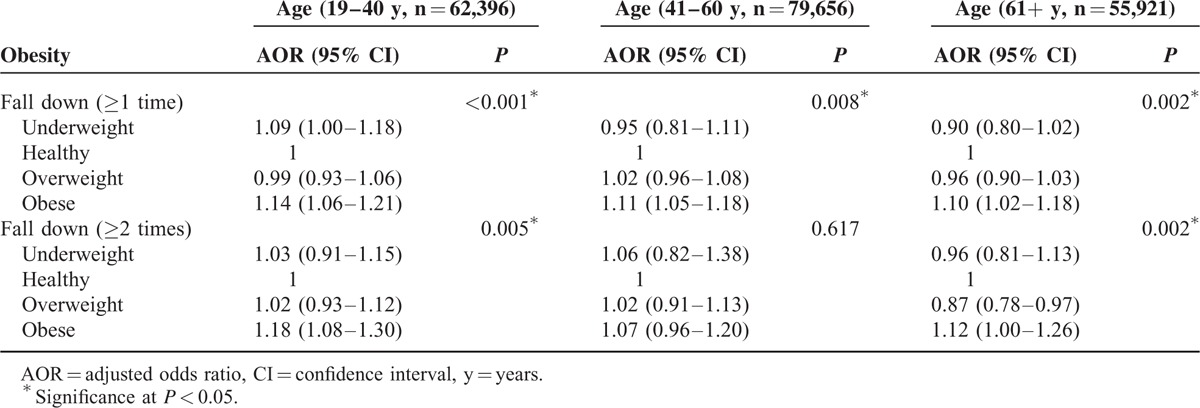
Subgroup Analysis of Obesity for Fall Down (≥1 Time Or ≥ 2 Times a Year) Using Multiple Logistic Regression Analysis With Complex Sampling (Model 2) According to Age Groups

In another subgroup analysis, the relationship between BMI and falls also differed according to outdoor or indoor falls (Table 4). Obese groups were significantly associated with outdoor falls but not with indoor falls in all age groups (each *P* < 0.05). The association between underweight and outdoor/indoor falls was inconsistent.

**TABLE 4:**
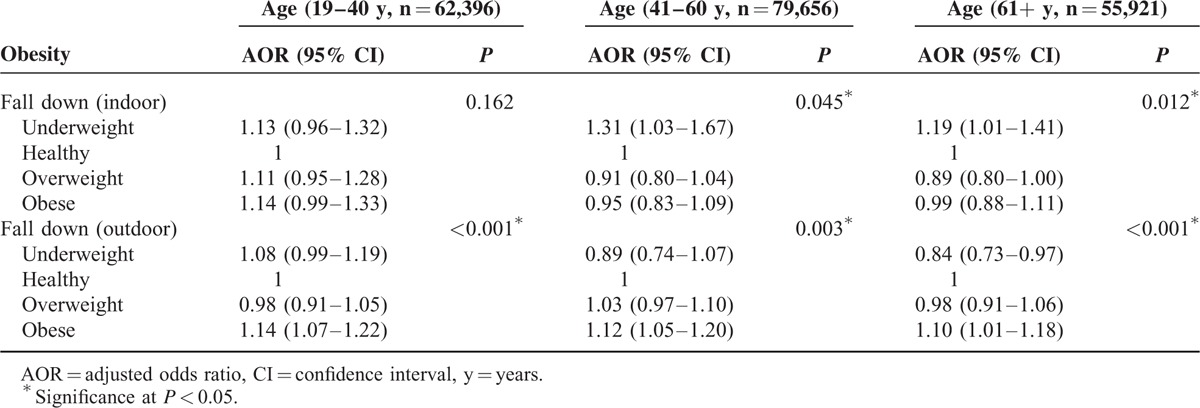
Subgroup Analysis of Obesity for Fall Down (≥1 Time a Year) Using Multiple Logistic Regression Analysis With Complex Sampling (Model 2) According to Age Groups and Location (Indoor or Outdoor)

## DISCUSSION

Consistent with previous studies, obese individuals were significantly associated with the increased risk of falls among Koreans when using Asian BMI classifications.^11,13^ Moreover, underweight status was also significantly positively associated with falls. However, there was no significant correlation between falls and overweight status. In the subgroup analysis, obese status was significantly associated with falls even in young adults, whereas there was only a significant relationship between falls and those who were underweight in young adults. When evaluating the association between the locations of falls and weight status, obesity was significantly associated with outdoor falls but did not display a significant association with indoor falls.

The obese group was significantly correlated with falls in the present study (AOR obese, ≥1 fall = 1.06, 95% CI = 1.02–1.10; AOR obese, ≥2 falls = 1.04, 95% CI = 0.99–1.10). This effect of obesity on falls may originate from the reduced physical ability among individuals who are obese. Previous studies have demonstrated that increased body weight adds pressure on the heels, which compromises postural stability and balance ability.^26^ In addition to this direct effect resulting from obesity, comorbid or complicated physiologic changes in people who are obese may also contribute to falls. Increased fat may result in the loss of muscle strength, which is referred to as “dynapenic obesity,” and is known to contribute to an increased risk of falls.^27^ Dynapenic obesity is also associated with limited mobility.^28^ In addition, increased adipose tissue also causes loss of muscle mass, which is termed “sarcopenic obesity.”^6^ Sarcopenia is linked with an increased risk of falls because of postural instability and reduced physical activity.^29–31^ Furthermore, it has been reported that sarcopenia is positively associated with a loss of bone mineral density and osteoporosis, leading to an increased fracture risk in older adults.^32^ These adverse relationships between obesity and dynapenia/sarcopenia/osteoporosis increase as individuals age.^33,34^ Because previous studies have primarily focused on the relationship between obesity and falls in older adults, our data move the field forward by identifying a relationship between falls and obesity in all adult groups, not exclusively older adults. It is also possible that other comorbid conditions of obesity such as heart disease, diabetes, sedentary behavior, and mood disorders may partially contribute to the relationship between obesity and falls.^35^ No significant correlations were identified between overweight status and falls in our data. However, our overweight group was based on the Asian BMI classification system that comprised subjects with BMI values of 23 ≤ BMI < 25, which is a range categorized as a healthy BMI in the WHO BMI classification system. In addition, our obese group (≥25) included the overweight category (25 ≤ BMI <30) of the WHO BMI classification system. Therefore, our results indicate that obesity was associated with falls even in the relatively lower Asian BMI classifications compared to the WHO classifications.

Interestingly, we identified a significant association between falls and underweight status (AOR underweight, ≥1 fall = 1.12, 95% CI = 1.05–1.19; AOR underweight, ≥2 falls = 1.14, 95% CI = 1.04–1.26). This may be explained by the fact that becoming underweight is often a result of an insufficient nutritional supply, which may also cause sarcopenia.^36–38^ Unlike the obesity-induced loss of muscle mass, being underweight leads to the loss of muscle mass without replacement by fat. The physical weakness from lost muscle mass combined with the fragility in extremely thin subjects could induce falls in the underweight. In line with this hypothesis, only a few previous studies conducted with underweight subjects suggested an increased risk of falls in thin subjects.^17,39^ Although other previous studies did not show a significant relationship between underweight status and falls, these studies were confined to the elderly or only included a small portion of the study population classified as underweight.^11,40^

We also identified some differences in the relationship between BMI and falls according to age group; few previous studies have focused on falls experienced by adults under age 60. In the subgroup analysis, although obese status was significantly associated with falls in all of the groups, underweight status was only significantly related to falls in young adults. The insignificant correlation between underweight status and falls in the middle-aged and older adult groups might be a result of the decreased statistical power due to the small number of underweight subjects in this study; underweight individuals accounted for only 5.6% of the enrolled participants, despite the large sample population (197,973 participants). It is also possible that some unknown underlying factors/mechanisms contribute to these differing results between young adults and adults. For instance, the reasons for falling down might differ based on age and obesity. According to the supplemental data (Supplement 1), falls experienced by young underweight adults occurred predominantly outdoors, with a relatively low rate of indoor falls.

The relationship between BMI and falls also differed according to the locations of the falls. Obesity was significantly associated with outdoor falls in all age groups, but there was no significant correlation between obesity and indoor falls. Because outdoor falls are generally associated with more external stimuli and activities than indoor falls, the adverse effects of obesity on mobility and stability might have more of an effect on outdoor falls than indoor falls. Moreover, outdoor falls (n = 27,587; 13.9%) were more frequent than indoor falls (n = 8098, 4.1%) in this study, which could have affected the differences in significance between indoor and outdoor falls among various groups.

Although we conducted a stratified statistical analysis based on the large representative population, we also conducted an analysis for ≥1 fall and a second analysis for ≥2 falls per year to minimize the influence of incidental falls regardless of susceptibilities in the data. We also adjusted for various confounding factors, but the results of our data had some limitations. In the subgroup analyses, the subgrouping of the study population resulted in a smaller number of analyzed subjects, which might attenuate the statistical power. For the obesity parameters, the BMI value was based on a weight-height ratio, which may not accurately represent the amounts of abdominal adipose tissue. In particular, the representativeness of the BMI value for obesity in older adults is limited.^41^ In addition, there was a possibility of recall bias because the present study was based on self-reported experiences of falls. Finally, cross-sectional study designs have an inherent limitation regarding the ability to establish a causal relationship between BMI groups and falls. Further studies with more representative and various parameters for underweight and obese using prospective designs will help overcome the present limitations.

## Supplementary Material

Supplemental Digital Content

## References

[1] Stewart Williams J Kowal P Hestekin H Prevalence, risk factors and disability associated with fall-related injury in older adults in low- and middle-incomecountries: results from the WHO Study on global AGEing and adult health (SAGE). *BMC Med* 2015; 13:147. 26099794 10.1186/s12916-015-0390-8 PMC4495610

[2] Murray CJ Vos T Lozano R Disability-adjusted life years (DALYs) for 291 diseases and injuries in 21 regions, 1990–2010: a systematic analysis for the Global Burden of Disease Study 2010. *Lancet* 2012; 380:2197–2223. 23245608 10.1016/S0140-6736(12)61689-4

[3] Ambrose AF Cruz L Paul G Falls and fractures: a systematic approach to screening and prevention. *Maturitas* 2015; 82:85–93. 26255681 10.1016/j.maturitas.2015.06.035

[4] Choi EJ Kim SA Kim NR Risk factors for falls in older Korean adults: the 2011 Community Health Survey. *J Korean Med Sci* 2014; 29:1482–1487. 25408578 10.3346/jkms.2014.29.11.1482 PMC4234914

[5] Ambrose AF Paul G Hausdorff JM Risk factors for falls among older adults: a review of the literature. *Maturitas* 2013; 75:51–61. 23523272 10.1016/j.maturitas.2013.02.009

[6] Hita-Contreras F Martinez-Amat A Cruz-Diaz D Osteosarcopenic obesity and fall prevention strategies. *Maturitas* 2015; 80:126–132. 25533145 10.1016/j.maturitas.2014.11.009

[7] Baker DI King MB Fortinsky RH Dissemination of an evidence-based multicomponent fall risk-assessment and -management strategy throughout a geographic area. *J Am Geriatr Soc* 2005; 53:675–680. 15817016 10.1111/j.1532-5415.2005.53218.x

[8] Blair SN Physical inactivity: the biggest public health problem of the 21st century. *Br J Sports Med* 2009; 43:1–2. 19136507

[9] Leischik R Foshag P Strauss M Physical activity, cardiorespiratory fitness and carotid intima thickness: sedentary occupation as risk factor for atherosclerosis and obesity. *Eur Rev Med Pharmacol Sci* 2015; 19:3157–3168. 26400517

[10] Jeon BJ The effects of obesity on fall efficacy in elderly people. *J Phys Ther Sci* 2013; 25:1485–1489. 24396217 10.1589/jpts.25.1485 PMC3881484

[11] Himes CL Reynolds SL Effect of obesity on falls, injury, and disability. *J Am Geriatr Soc* 2012; 60:124–129. 22150343 10.1111/j.1532-5415.2011.03767.x

[12] Lin HW Bhattacharyya N Impact of dizziness and obesity on the prevalence of falls and fall-related injuries. *Laryngoscope* 2014; 124:2797–2801. 24986701 10.1002/lary.24806

[13] Ren J Waclawczyk A Hartfield D Analysis of fall injuries by body mass index. *South Med J* 2014; 107:294–300. 24937728 10.1097/SMJ.0000000000000097

[14] Deurenberg-Yap M Schmidt G van Staveren WA The paradox of low body mass index and high body fat percentage among Chinese, Malays and Indians in Singapore. *Int J Obes Relat Metab Disord* 2000; 24:1011–1017. 10951540 10.1038/sj.ijo.0801353

[15] Deurenberg P Deurenberg-Yap M Guricci S Asians are different from Caucasians and from each other in their body mass index/body fat per cent relationship. *Obes Rev* 2002; 3:141–146. 12164465 10.1046/j.1467-789x.2002.00065.x

[16] Taskforce IO The Asia-Pacific perspective: Redefining obesity and its treatment. 2000 http://www.wpro.who.int/nutrition/documents/docs/Redefiningobesity.pdf Accessed November 20, 2014.

[17] Honeycutt PH Ramsey P Factors contributing to falls in elderly men living in the community. *Geriatr Nurs* 2002; 23:250–255. 12386601 10.1067/mgn.2002.128785

[18] Rim H Kim H Lee K Validity of self-reported healthcare utilization data in the Community Health Survey in Korea. *J Korean Med Sci* 2011; 26:1409–1414. 22065895 10.3346/jkms.2011.26.11.1409 PMC3207042

[19] Oh DH Kim SA Lee HY Prevalence and correlates of depressive symptoms in korean adults: results of a 2009 korean community health survey. *J Korean Med Sci* 2013; 28:128–135. 23341723 10.3346/jkms.2013.28.1.128 PMC3546091

[20] Hah JH Sim S An SY Evaluation of the prevalence of and factors associated with laryngeal diseases among the general population. *Laryngoscope* 2015; 125:2536–2542. 26154733 10.1002/lary.25424

[21] Kim SY Kim SG Sim S Excessive sleep and lack of sleep are associated with slips and falls in the adult Korean population: a population-based cross-sectional study. *Medicine* 2016; 95:e2397. 26825881 10.1097/MD.0000000000002397 PMC5291551

[22] Kim SY Sim S Kim SG Sleep deprivation is associated with bicycle accidents and slip and fall injuries in Korean adolescents. *PloS One* 2015; 10:e0135753. 26280345 10.1371/journal.pone.0135753 PMC4539229

[23] Kong IG Lee HJ Kim SY Physical activity, study sitting time, leisure sitting time, and sleep time are differently associated with obesity in Korean adolescents: a population-based study. *Medicine* 2015; 94:e1965. 26554807 10.1097/MD.0000000000001965 PMC4915908

[24] Development OfEC-oa. What are equivalence scales? 2009 http://www.oecd.org/eco/growth/OECD-Note-EquivalenceScales.pdf Accessed November 20, 2014.

[25] Organization WH. The International Classification of adult underweight, overweight and obesity according to BMI. 2004 http://apps.who.int/bmi/index.jsp?introPage=intro_3.html Accessed March 2, 2015.

[26] Clark BC Manini TM Functional consequences of sarcopenia and dynapenia in the elderly. *Curr Opin Clin Nutr Metab Care* 2010; 13:271–276. 20154609 10.1097/MCO.0b013e328337819e PMC2895460

[27] Scott D Sanders KM Aitken D Sarcopenic obesity and dynapenic obesity: 5-year associations with falls risk in middle-aged and older adults. *Obesity (Silver Spring)* 2014; 22:1568–1574. 24585708 10.1002/oby.20734

[28] Bouchard DR Janssen I Dynapenic-obesity and physical function in older adults. *J Gerontol A Biol Sci Med Sci* 2010; 65:71–77. 19887536 10.1093/gerona/glp159

[29] Zamboni M Mazzali G Fantin F Sarcopenic obesity: a new category of obesity in the elderly. *Nutr Metab Cardiovasc Dis* 2008; 18:388–395. 18395429 10.1016/j.numecd.2007.10.002

[30] Kim SH Kim TH Hwang HJ The relationship of physical activity (PA) and walking with sarcopenia in Korean males aged 60 years and older using the Fourth Korean National Health and Nutrition Examination Survey (KNHANES IV-2, 3), 2008–2009. *Arch Gerontol Geriatr* 2013; 56:472–477. 23298535 10.1016/j.archger.2012.12.009

[31] Hida T Harada A Fall risk and fracture. Diagnosing sarcopenia and sarcopenic leg to prevent fall and fracture: its difficulty and pit falls. *Clin Calcium* 2013; 23:707–712. 23628684

[32] Cho WS Kim JE Kim CH Long-term outcomes after combined revascularization surgery in adult moyamoya disease. *Stroke* 2014; 45:3025–3031. 25184359 10.1161/STROKEAHA.114.005624

[33] Baumgartner RN Body composition in healthy aging. *Ann N Y Acad Sci* 2000; 904:437–448. 10865787 10.1111/j.1749-6632.2000.tb06498.x

[34] Kim TN Yang SJ Yoo HJ Prevalence of sarcopenia and sarcopenic obesity in Korean adults: the Korean sarcopenic obesity study. *Int J Obes (Lond)* 2009; 33:885–892. 19564878 10.1038/ijo.2009.130

[35] Mitchell RJ Lord SR Harvey LA Obesity and falls in older people: mediating effects of disease, sedentary behavior, mood, pain and medication use. *Arch Gerontol Geriatr* 2015; 60:52–58. 25307955 10.1016/j.archger.2014.09.006

[36] Tinetti ME Doucette J Claus E Risk factors for serious injury during falls by older persons in the community. *J Am Geriatr Soc* 1995; 43:1214–1221. 7594154 10.1111/j.1532-5415.1995.tb07396.x

[37] Simon JA Mack CJ Prevention and management of osteoporosis. *Clin Cornerstone* 2003; 5 suppl 2:S5–S12. 15035554 10.1016/s1098-3597(03)90042-1

[38] Lee JS Auyeung TW Kwok T Associated factors and health impact of sarcopenia in older chinese men and women: a cross-sectional study. *Gerontology* 2007; 53:404–410. 17700027 10.1159/000107355

[39] O’Neil CA Krauss MJ Bettale J Medications and patient characteristics associated with falling in the hospital. *J Patient Saf* 2015; [Epub ahead of print]. 10.1097/PTS.0000000000000163 PMC4573384 25782559

[40] Koepp GA Snedden BJ Levine JA Workplace slip, trip and fall injuries and obesity. *Ergonomics* 2015; 58:674–679. 25532054 10.1080/00140139.2014.985260

[41] ACKD. Definition and epidemiology of obesity. *Korean Assoc Med J Edet* 2004; 289–297.[Epub ahead of print].

