# Supplementary material for: Association Between Obesity and Falls Among Korean Adults: A Population-Based Cross-Sectional Study
Source: Medicine (Baltimore). 2016 Mar 25;95(12):e3130. doi: 10.1097/MD.0000000000003130 (PMC4998385; doi:10.1097/MD.0000000000003130)
Supplement: Supplemental Digital Content [file medi-95-e3130-s001.docx]

Supplement 1. Rates of fall down at indoor and outdoor according to age group and obesity.

| Age group | **Indoor fall down (%)** | | | |
| --- | --- | --- | --- | --- |
|  | Underweight | Healthy weight | Overweight | Obese |
| Age 19-40 y | 4.6 | 3.3 | 2.8 | 2.7 |
| Age 41-60 y | 3.1 | 2.7 | 2.7 | 2.9 |
| Age 61+ y | 9.5 | 6.6 | 5. | 7.0 |
|  | **Outdoor fall down (%)** | | | |
|  | Underweight | Healthy weight | Overweight | Obese |
| Age 19-40 y | 17.1 | 15.1 | 14.5 | 16.6 |
| Age 41-60 y | 11.6 | 11.5 | 11.7 | 13.3 |
| Age 61+ y | 14.8 | 14.4 | 14.8 | 16.1 |
